# Primary Care Physicians’ Knowledge, Attitude, and Potential Referral Barriers towards Bariatric Surgery: A Northern Saudi Study

**DOI:** 10.3390/medicina58121742

**Published:** 2022-11-28

**Authors:** Anfal Mohammed Alenezi, Ashokkumar Thirunavukkarasu, Abdulaziz Khalid Alrasheed, Talal Ahmed Alsharari, Khalid Bsam A. Almadhi, Malek Mohammed N. Almugharriq, Ragad Ahmed Alshalan, Khalid Muteb Alshalan, Abdullah Alturqi Kurdi Alanazi, Wesam Sultan Albayyali

**Affiliations:** 1 Department of Surgery, College of Medicine, Jouf University, Sakaka 72388, Saudi Arabia; 2 Department of Community and Family Medicine, College of Medicine, Jouf University, Sakaka 72388, Saudi Arabia; 3 Department of General Surgery, King Saud Medical City, Riyadh 12745, Saudi Arabia; 4 College of Medicine, Jouf University, Sakaka 72388, Saudi Arabia

**Keywords:** bariatric surgery, referral barriers, primary care, morbid obese, complications

## Abstract

*Introduction:* Bariatric surgery is the most effective procedure for sustained weight loss and control of obesity-associated comorbidities among morbidly obese patients. Successful bariatric surgery depends on a multidisciplinary approach involving all healthcare workers, including the primary care physicians, from the referral of patients to long-term follow-up. The present study assessed the knowledge, attitude, and potential referral barriers of primary care physicians to bariatric surgery and associated sociodemographic factors. *Materials and methods:* The present analytical cross-sectional study was conducted among 280 randomly selected primary care physicians using a standard and validated data collection tool. We performed an independent t-test and one-way ANOVA to find the association between sociodemographic characteristics and knowledge, attitude, and referral barrier scores. Furthermore, multilinear regression analysis was executed to determine the association among knowledge, attitude, and barriers. *Results:* The current study found that 52.9%, 19.3%, and 59.3% had a low score in the knowledge, attitude, and barriers categories. The attitude scores were significantly associated with the education status (*p* = 0.005) and current position at primary health centers (*p* = 0.012), and the referral barriers score was significantly associated with the work experience duration (*p* = 0.004). We found a positive relationship between knowledge and attitude (regression coefficient (β) [95% CI]:0.389 [0.154 to 0.585], *p* = 0.001) and a negative relationship between knowledge and referral barriers (β [95% CI]: −0.291 [−0.127 to −0.058], *p* = 0.007). *Conclusions:* Our survey findings suggest that a lack of knowledge regarding bariatric surgery led to several concerns and referral barriers among the physicians. Therefore, the recommendation is to improve the primary care physicians’ knowledge through continuing medical education, symposium, and other suitable training methods with a special focus on obesity care in the curriculum. Furthermore, a mixed-method survey involving other provinces of the KSA is warranted to formulate the region-specific training needs.

## 1. Introduction

Obesity has become a global epidemic of concern and is regarded as a tremendous present-day public health challenge [1,2]. Obesity prevalence is increasing considerably in middle and low-income countries, as stated by the World Health Organization (WHO) [1]. Overweight and obese patients are at increased risk for developing many health problems, including type 2 diabetes mellitus (T2DM), hypertension, coronary heart disease, dyslipidemia, osteoarthritis, and numerous psychosocial issues. According to the WHO, the prevalence rate of obesity in Saudi Arabia (KSA) is the fastest-growing rate in the world [3]. A nationwide study carried out by Althumiri et al. in 2021 in the KSA revealed that the prevalence of obesity was 24.7% among the adult population [4]. Some of the other authors also revealed a high prevalence of overweight and obesity in the KSA, suggesting the failure of lifestyle and pharmacological methods to control the obesity epidemic [5,6]. Bariatric surgery is a well-established procedure carried out among morbid/severe obese patients (BMI 35 kg/m^2^ and above) by altering the digestive system [7,8]. Bariatric surgery is the most effective procedure for sustained weight loss, control of obesity-associated comorbidities, increase in quality of life, and decrease in mortality [9,10]. Currently, this is the only modality available for managing morbid obesity patients, as reported by some authors [11,12]. Successful bariatric surgery is dependent on the multidisciplinary approach involving all healthcare workers, including primary care physicians, from the referral of patients to long-term follow-up [13]. Primary care is the first level of contact with the health services provided in primary health centers (PHCs).

The primary care physicians working at PHCs ensure that the public receives comprehensive and quality care, including health promotion, prevention, treatment, and long-term post-surgical follow-up care [14]. Several studies reported that follow-up among post-bariatric surgical patients after 5 to 10 years is very low [15,16]. This indicates the importance of physicians at a PHC knowing how to handle the possible adverse effects among the post-bariatric surgical patients’ long-term follow-up [15,17]. In the KSA, all healthcare services, including bariatric surgery, are provided free of cost for all citizens and expatriates working in the public sectors. However, they must be initially screened by the physicians at primary health centers (PHCs), as a PHC is the first level of contact for all eligible obese patients. Therefore, the continuous evaluation of knowledge and attitude of primary care physicians towards bariatric surgery and its importance in obesity management is essential to strengthen the health services related to bariatric surgery, as they form the core members of the treatment of morbidly obese patients [10,15,18].

A study conducted by Memerian et al. in 2021 among Swedish family medicine doctors revealed that high concerns related to bariatric surgeries and their complications are associated with the poor referral of morbidly obese patients [19]. They conducted a web-based cross-sectional survey among 1100 physicians using a structured questionnaire that assessed participants’ knowledge, attitude, and postoperative concerns. Furthermore, Memerian et al. executed Spearman’s analysis and regression analysis to find the association between knowledge, attitude, and barriers. Another study conducted by Fan M et al. revealed poor acceptance of bariatric surgery for obesity management among Chinese nurses [20]. Fan M et al. reported the findings from the data analysis using a validated and pretested tool. An electronic survey conducted among multicentric community hospital primary physicians in the USA found that physicians have a positive attitude towards the referral of bariatric patients [21]. The findings of their study used a five-point Likert scale questionnaire to assess the attitudes and referral barriers of participants to patients eligible for bariatric surgery. From our extensive literature search from major databases, we found that limited data are available in the KSA, especially from the northern KSA. Hence, this study assessed the knowledge, attitude, and referral barriers toward bariatric surgery and associated sociodemographic factors among primary care physicians. Furthermore, we evaluated the correlation between knowledge, attitude, and referral barriers.

## 2. Materials and Methods

### 2.1. Study Description

This primary health center based cross-sectional study was implemented in the northern region of the KSA from March 2022 to August 2022. We have included four areas: Hail, Aljawf, Tabuk, and Arar. The ministry of health controls all PHCs, and 943 primary care physicians working in these selected regions are distributed in 185 PHCs.

### 2.2. Sample Size and Method

**Figure 1:**
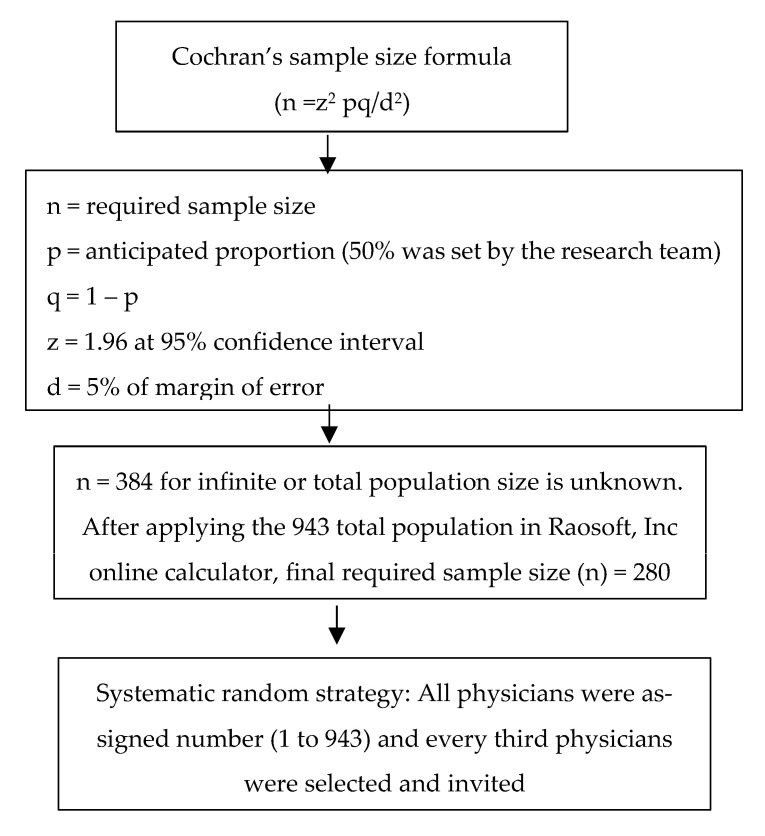
Sampling strategy flow chart.

The minimum number of participants needed for this survey was estimated based on Cochran’s equation (*n* = z^2^ pq/d^2^) [22]. Regarding the calculation of the equation, the anticipated proportion is presented by p, 1 − p is q, and the margin of error at 5% is d. While estimating the required number of participants, the factors included were anticipated proportion (p) at 50%, power at 80%, and level of confidence at 95%. Since there is no previous study from our region in this context, we considered p as 50% as a rule of thumb. Applying these components to Cochran’s formula, followed by calculating the required sample size for the total primary care physicians’ number of 943 (finite population), we concluded that 280 is the size of the sample needed for the present study. We performed a systematic sampling technique (representative method) to choose the eligible physicians. In this method, we assigned a number to all the physicians and arranged them in ascending order in Statistical Package for Social Science (SPSS) software. This was considered as the sampling frame for the present study. The research team selected every 3rd participant from the sampling frame by applying this sampling technique and invited them to participate in the survey. Figure 1 summarizes the sampling strategy.

### 2.3. Inclusion and Exclusion Criteria

The research team included all primary care physicians from the selected regions. We excluded the participants on leave, those who denied participation in the survey, those who work in other government settings (general and tertiary care hospitals), and the primary care physicians who work in private settings.

### 2.4. Data Collection

We initiated the survey after getting ethical clearance from the local committee for bioethics (LCBE), Jouf University (Wide approval no: 2-09-043). The data collectors explained the study to the physicians, and those who were willing to participate through informed consent were requested to complete the approved, self-administered survey form (Google form) on the electronic devices of the research team at the participant’s workplace. The data collection form (Survey questionnaire — Appendix A) was designed by the research team from open-source questionnaires adapted from previously published studies [19,21] and inputs from the panel of experts (bariatric surgeons and family medicine consultants) working in the KSA. To assure validity and reliability, we followed the following steps: reviewing the designed questionnaire by experts in the family medicine/primary care and surgery department through a focus group discussion, then executing a pilot study (pretest) among thirty primary care physicians in the local settings. The pilot study’s target population confirmed that the questionnaire used was simple, easy to follow, clear to understand, and suitable for local settings. Lastly, no missing data were detected in the used questionnaires of the pilot study. The Cronbach’s alpha values (a measure of internal consistency) of the knowledge, attitude, and referral barriers were 0.77, 0.81, and 0.76, respectively. Hence, we collected the data using this reliable, validated, and locally suitable questionnaire. The data collection form did not request the participants to give their personal identification details. Only the principal investigator was given the authorization to access, download, and convert the data for analysis. Hence, the confidentiality and anonymity of the survey are maintained.

Participants’ sociodemographic characters were described in the first section of the questionnaire, including gender, age, marital status, education level, nationality, and work experience duration. The second part of the questionnaire was composed of eight questions in the knowledge category and eight in each attitude and potential barrier category. Knowledge category questions were related to bariatric surgery indications, complications, and follow-up, and they were presented in multiple-choice questions format. We gave one point for the correct answer and zero for the wrong answer for all knowledge-related questions. Primary healthcare physicians responded on a 5-point Likert scale as “strongly agree”, “agree”, “neutral”, “disagree” and “strongly disagree”, in the attitude and potential barrier scales. These 5-point Likert scales were given scores 5, 4, 3, 2, and 1, respectively. Based on the responses in each section (knowledge, attitude, and potential barriers), we computed the participants’ total scores in each section. Finally, we calculated the mean ± standard deviation (SD) for knowledge, attitude, and potential barriers for further analysis to find the association with the sociodemographic characteristics. Regarding the interpretation of the knowledge, attitude, and barriers scores, it was described as high when it was 80% or above, medium when it was 60% to 79%, and low when it was less than 60%.

### 2.5. Statistical Analysis

We used the Statistical Package for Social Sciences program (SPSS, Version 21.0) for exporting data from the spreadsheet, coding, and recoding for all variables and necessary analysis. Qualitative data variables are presented as frequency (*n*) and percentage, while mean ± SD is used for continuous data presentation. We checked and confirmed the normality assumption of the data through the Kolmogorov–Smirnoff test. Therefore, the research team applied one-way analysis of variance (ANOVA) and independent *t*-test to find the association between the knowledge, attitude, and referral barriers scores with sociodemographic details. To assess the strength and direction of association between knowledge, attitude, and referral barriers scores, a Pearson’s correlation test was done. Lastly, multilinear regression analyses (after adjusted with covariables) were performed to detect the association between knowledge, attitude, and barriers scales. All the statistical tests in the present study were 2-tailed, and a *p*-value that was lower than 0.05 was shown as statistically significant.

## 3. Results

Of the 280 responded physicians, the majority (58.6%) were males, belonging to the age group (31–44 years), non-Saudi nationals (55.7%), and with work experience of less than five years (55.7%) (Table 1).

**Figure 2:**
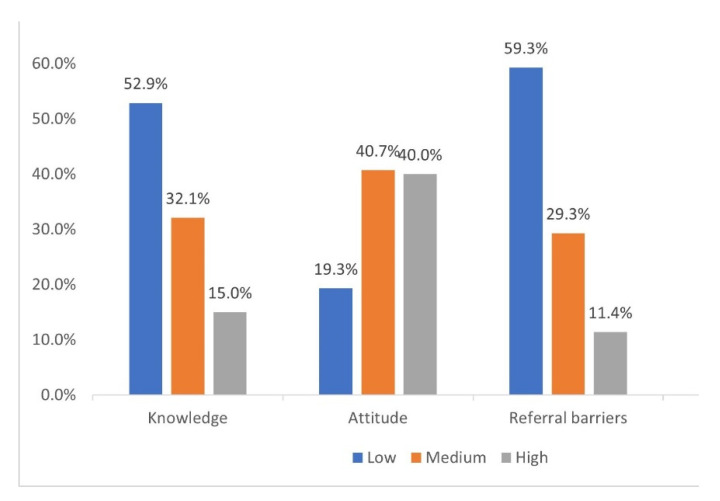
Knowledge, attitude, and referral barriers subcategories (*n* = 280).

The present study categorized the knowledge, attitude, and referral barriers score as per Bloom’s criteria. Of the population studied, 52.9%, 19.3%, and 59.3% had a low score on the knowledge, attitude, and barriers scales, respectively (Figure 2).

Table 2 shows the relationship between knowledge, attitude, referral barriers scores, and background characteristics. Of the 280 respondents, the present study did not find any significant association of the knowledge score with the sociodemographic characteristics of the primary care physicians. Education level (*p* = 0.005) and professional cadre at PHC (*p* = 0.012) were significantly associated with attitude scores, while the referral barriers score was significantly associated with the work experience duration (*p* = 0.004). 

Table 3 depicts that nearly half of the participated primary care physicians either strongly agreed or agreed with the statements related to the barriers for referring eligible patients for bariatric surgery. Of the 280 primary care physicians, 168 (60%) either strongly agreed or agreed that they were concerned about the perioperative risks during bariatric surgical procedures, and 160 (57.1%) considered that the costs for bariatric surgery are a burden for society and healthcare system.

A positive correlation between knowledge and attitude scales was identified in the Pearson’s correlation analysis (correlation coefficient: 0.357, *p* < 0.001) and a negative correlation between the knowledge and referral barrier scales (correlation coefficient: −0.291, *p* < 0.001) (Table 4). Using the multilinear regression analysis, we found a positive relationship between knowledge and attitude (regression coefficient (β) [95% CI]: 0.389 [0.154 to 0.585], *p* = 0.001) and a negative relationship between knowledge and referral barriers (β [95% CI]: −0.291 [−0.127 to −0.058], *p* = 0.007) (Table 5).

## 4. Discussion

Bariatric surgical management of morbidly obese and patients with metabolic disorders is gaining popularity in the KSA. However, this is still a new discipline for several healthcare professionals. Thus, this research was conducted to assess primary care physicians’ knowledge, attitude, and referral barriers towards bariatric surgery.

Healthcare delivery is a knowledge-guided discipline, and the primary care physicians who work in PHCs must have adequate and organized knowledge of bariatric surgery [23,24]. This can enable them to search for eligible patients to refer to bariatric surgery that might permanently change the life of morbidly obese patients for the better. More than half of the primary care physicians (52.9%) in the present study had insufficient knowledge regarding bariatric surgery. These results demonstrate that primary care physicians were deficient in knowledge of bariatric surgery and were required to be more proactive in recommending surgical options for eligible candidates according to the criteria. We did not find a significant association between knowledge and the participants’ sociodemographic characteristics. This further explains the need for training programs for all primary care physicians, regardless of their background characteristics. In contrast, a study carried out among Turkish primary care physicians revealed a significant association with the young primary care physicians (*p* < 0.05) [25]. Similarly to our findings, a recent survey completed in Saudi Arabia by AlDhaban D et al. noted low knowledge and unfamiliarity with bariatric surgery among primary care physicians [26]. A survey conducted among family doctors in Ontario by Auspitz et al. found a knowledge and awareness gap among family doctors regarding bariatric surgery for managing morbidly obese patients [27]. In contrast, another survey conducted in Riyadh city, KSA, by Alwhibi MW et al. found that more than half of the physicians reported favorable knowledge of bariatric surgery [28]. The possible differences between our study and the latter study might be due to the inclusion of primary care physicians. We included the participants working at PHCs only, while Alwhibi MW et al. selected the physicians serving in PHCs and medical city (apex hospital). Interestingly, a Swedish survey reported that nearly three-quarters of family doctors and primary care physicians had good knowledge regarding referral of the patients for bariatric surgery [19]. These wide variations in the bariatric-surgery-related knowledge and its associated factors across studies ensured that the present study and its findings are essential in planning region-specific training needs.

A positive attitude based on scientific facts is essential in primary care, as the attitude is contagious; it will help patients to choose the appropriate treatment regimen for their obesity management [29]. However, the current study found that 40% of the primary care physicians had a positive (high) attitude towards bariatric surgery. In contrast, the remainder had a low or medium level of attitude, which is significantly associated with the education status and professional cadre. The present study findings indicate that more than half of the primary care physicians’ attitudes were suboptimal, which could hinder them from referring eligible patients for necessary management. Hence, improving physicians’ attitudes could be critical in referring patients to bariatric surgical treatment. Our study findings are similar to a survey by Memiarian E et al. and AlDhaban D et al. conducted in 2021. In their study, less than half of the participants had a positive attitude towards bariatric surgery [19]. In contrast to our findings and other studies carried out in Saudi Arabia by AlDhaban D et al., research conducted in the USA by Sarwer DB et al. found that physicians had a favorable attitude towards bariatric surgery and referring obesity and T2DM patients [30]. These dissimilarities between our study and Sarwer DB et al.’s findings could be differences in the included participants. The present study included only primary care physicians, whereas Sarwar DB et al. included primary care/family medicine doctors and endocrinologists.

Barriers in the utilization of healthcare services, including bariatric services, are classified into different levels: organizational, care provider, and individual levels. Of all barriers, healthcare providers play a significant role in removing the obstacles to referring eligible patients for bariatric surgical management [31,32]. However, the current study found that more than half of the primary healthcare physicians had barriers in several categories. The barriers were significantly higher among the primary care physicians with work experience of less than five years (mean ± SD = 19.29 ± 5.80, *p* = 0.004). The present study also found that the physicians were concerned with bariatric surgery’s perioperative and postoperative complications. More than one-fourth of the participants were not confident enough with the post-bariatric surgical patient follow-up. This indicates that in the present study, primary care physicians were unaware of the exact proportion of these potential complications, and the concept benefits outweigh the risks [9]. Similar to the current research, a survey conducted in 2021 by Conaty EA et al. reported that concerns about post-surgical risks were the main hindering factors to referring the patients for bariatric surgery among the primary care physicians who participated in their research [21]. Our study’s findings are supported by another research conducted in the KSA by Alzuhgbi et al. [33]. In their study, low perceived benefits and safety concerns were commonly reported among the participants.

The univariate and regression analysis of the present study explored a positive association between knowledge and attitude (β = 0.389, *p* = 0.001) and a negative association between knowledge and referral barriers (β = −0.291, *p* = 0.007). Despite the available evidence on the effectiveness of bariatric surgery, inadequate knowledge could lead to more concerns and barriers to referring eligible patients. The present study’s findings are supported by a survey conducted in 2021 by Memarian E et al. among primary care physicians. They found a significant positive correlation between high knowledge with attitude and a negative correlation with the concerns and referral patterns [19]. These results from the current study indicate that adequate knowledge will significantly impact physicians’ attitudes toward bariatric surgery. Furthermore, the present study results identified that attitudes and potential referral barriers assist in identifying the gaps in primary care physicians’ knowledge that may be aiming for necessary intervention.

## 5. Strengths and Weaknesses of the Present Study

The present study assessed primary care physicians’ knowledge, attitude, and referral barriers using a standard methodology and validated data collection tool. The present study also used a proper sampling method to select them. However, the readers have to consider specific concerns while interpreting this research paper. Firstly, we used a cross-sectional study that evaluated the association, not the causation. Next, the possibility of biases associated with the self-reported survey cannot be excluded. Furthermore, the results of this study may not be generalized to physicians working in other health facilities such as general hospitals and specialty hospitals. Finally, we explored the primary care physicians’ potential referral barriers through a closed-end questionnaire designed by the research team. Hence, the present study could not find all potential barriers perceived by the primary care physicians.

## 6. Conclusions

This study revealed that more than half of primary healthcare physicians had low knowledge regarding bariatric surgery indications and complications. Their knowledge score is positively associated with their attitude towards bariatric surgery. The findings suggest that a lack of knowledge about bariatric surgery led to several concerns and referral barriers among physicians. Hence, addressing this knowledge gap is an essential component in improving obesity care and related complications, which is a significant public health issue in the KSA and other Middle East countries. Therefore, the recommendation is to improve the knowledge of primary care physicians through continuing medical education, workshops, symposiums, and other suitable training methods with a special focus on the care of bariatric surgery care in the curriculum. Furthermore, a multicentric mixed method survey involving other provinces of the KSA and primary care physicians working in other sectors is warranted to find potential new barriers that were not explored in this survey.

## Figures and Tables

**Table 1:** Participants’ background characteristics (*n* = 280).

Physicians’ Characteristics	Frequency (*n*)	%
Age category		
Up 30 years	100	35.7
31–44 years	106	37.9
45 years and above	74	26.4
Gender		
Male	164	58.6
Female	116	41.4
Highest education		
Bachelor (MBBS)	152	54.3
Masters (MD)	58	20.7
Saudi Board	42	15
Fellowship/PhD	28	10
Participants’ nationality		
Saudi	124	44.3
Non-Saudi	156	55.7
Work experience duration		
Up to 5 years	156	55.7
More than 5 years	124	44.3
Current professional cadre		
Resident	188	67.1
Specialist	66	23.6
Consultant	26	9.3

**Table 2:** Relationship between knowledge, attitude, and perceived referral barriers scores with the background characteristics (*n* = 280).

Variables	Knowledge		Attitude		Referral Barriers	
	Mean ± SD	*p*-Value	Mean ± SD	*p*-Value	Mean ± SD	*p*-Value
Age category *						
Up 30 years	5.44 ± 1,92		23.88 ± 4.62		19.42 ± 5.27	
31–44 years	5.47 ± 1.97	0.949	23.15 ± 5.86	0.175	17.83 ± 6.53	0.099
45 years and above	5.38 ± 1.82		24.59 ± 5.14		17.78 ± 6.05	
Gender **						
Male	5.34 ± 2.02	0.066	24.68 ± 5.03	0.713	18.87 ± 6.44	0.112
Female	5.57 ± 1.73		22.53 ± 5.04		17.71 ± 5.29	
Highest education *						
Bachelor (MBBS)	5.47 ± 2.08		24.32 ± 4.66		18.03 ± 5.89	
Masters (MD)	5.17 ± 1.72	0.464	21.76 ± 5.82	0.005 ***	18.90 ± 6.70	0.522
Saudi Board	5.76 ± 1,82		24.90 ± 5.14		18.14 ± 6.26	
Fellowship/PhD	5.29 ± 1.30		23.50 ± 5.22		19.64 ± 4.59	
Participants’ nationality **						
Saudi	5.52 ± 2.07	0.53	24.13 ± 4.87	0.325	18.68 ± 6.02	0.47
Non-Saudi	5.37 ± 1.77		23.53 ± 5.34		18.15 ± 6.00	
Work experience duration **						
Up to 5 years	5.33 ± 1.89	0.314	23.28 ± 5.37	0.058	19.29 ± 5.80	0.004 ***
More than 5 years	5.56 ± 1.92		24.44 ± 4.77		17.24 ± 6.09	
Current professional cadre *						
Resident	5.47 ± 1.84		24.29 ± 4.75		17.97 ± 5.85	
Specialist	5.39 ± 2.01	0.094	22.15 ± 5.99	0.012 ***	19.55 ± 5.86	
Consultant	5.31 ± 2.13		24.38 ± 4.81		18.46 ± 7.25	0.185

* One-way ANOVA, ** Independent samples *t* test, *** Significant value.

**Table 3:** Potential barriers to refer the morbid obese patients to the bariatric surgery centers (*n* = 280).

Barriers	Strongly Agree	Agree	Neutral	Disagree	Strongly Disagree
	*n* (%)	*n* (%)	*n* (%)	*n* (%)	*n* (%)
Most of my obese patients cannot afford bariatric surgery	46 (16.4)	82 (29.3)	72 (25.7)	62 (22.1)	18 (6.4)
Diet and exercise are effective means for sustained weight loss in morbid obese patients	52 (18.6)	94 (33.6)	62 (22.1)	62 (22.1)	10 (3.6)
I am not comfortable managing patients after surgery	26 (9.3)	80 (28.6)	62 (22.1)	78 (27.9)	34 (12.2)
I don’t feel confident to discuss with my patients as surgery is the treatment for morbid obese patients	28 (10.0)	56 (20.0)	46 (16.4)	118 (42.1)	32 (11.4)
I am concerned about the risk associated with the operation (e.g., anastomosis leakage, infection, bleeding)	68 (24.3)	100 (35.7)	70 (25.0)	30 (10.7)	12 (4.3)
I am concerned with the risk for postoperative complications	54 (19.3)	108 (38.6)	78 (27.9)	26 (9.3)	14 (5.0)
The long-term consequences of bariatric surgery are not completely known	36 (12.9)	90 (32.1)	84 (30.0)	48 (17.1)	22 (7.9)
The costs for bariatric surgery are a burden for society and healthcare system.	70 (25.0)	90 (32.1)	60 (21.4)	36 (12.9)	24 (8.6)

**Table 4:** Pearson’s correlation analysis between knowledge, attitude, and referral barriers.

	Pearson’s Coefficient Value (r)	*p*-Value
Knowledge—Attitude	0.357	<0.001 *
Knowledge—Referral barriers	−0.287	<0.001 *
Attitude—Referral barriers	0.012	0.846

* Statistically significant value.

**Table 5:** Relationship between knowledge, attitude, and potential referral barriers (multilinear regression analysis).

	Knowledge	
	Regression Coefficient (95% Confidence Interval)	*p*-Value
Attitude	0.389 (0.154 to 0.585)	0.001 *
Referral barriers	−0.291 (−0.127 to −0.058)	0.007 *

Adjusted variables: age category, gender, highest education, participants’ nationality, work experience duration, current professional cadre. * Statistically significant value.

## Data Availability

The corresponding author can make the data in the study available upon reasonable request.

## Appendix A

Dear doctor,

I appreciate your willingness to participate in the present research survey to assess the knowledge, attitude, and potential barriers among northern Saudi primary care physicians. The total duration to complete this survey may take around 10 minutes. For further clarifications related to this survey, you may contact the principal investigator Dr. Anfal Alenzi, Department of General Surgery, College of Medicine, Jouf University, Saudi Arabia.

Data collection proforma

Age (specify in years):

Gender:

1. Male
2. Female

Qualifications (Select the highest qualification):

1. Bachelor
2. Master
3. Saudi Board
4. Fellowship/PhD

Nationality:

1. Saudi
2. Non Saudi

Years working at PHC (Specify the years):

Position:

1. Resident
2. Specialist
3. Consultant

Next few questions will be related to your current awareness about bariatric surgery such as indications, types, prognosis, follow up and complications. You may choose the best answer as per your knowledge.

1. At what minimum body mass index (BMI) would you consider referring to a patient WITHOUT comorbidities (hypertension, diabetes mellitus, sleep apnea) to undergo bariatric surgery?
  - 30
  - 35
  - 40
  - 45
2. At what minimum BMI would you consider referring a patient WITH comorbidities (hypertension, diabetes mellitus, sleep apnea) to undergo bariatric surgery?
  - 30
  - 35
  - 40
  - 45
3. Remission of type II diabetes mellitus occurs in what percentage of patients who have undergone bariatric surgery?
  - 20%-40%
  - 60%
  - ≥80%
  - I do not know
4. How long is a typical hospital stay for a patient undergoing laparoscopic bariatric surgery?
  - 24–48 hours
  - 3–5 days
  - One week
  - I do not know
5. What is the mortality rate associated with bariatric surgery?
  - <1%
  - 2%
  - 6%
  - >10%
  - I do not know
6. Remission of diabetes is a known benefit of bariatric surgery. Approximately how long after surgery will patients see this benefit?
  - Days after surgery
  - 6 months after surgery
  - Improvement of diabetes mirrors weight loss
  - Improvement of diabetes occurs after significant weight loss has been achieved and maintained
7. Common postoperative side effects of bariatric surgery include.
  - Dumping syndrome
  - Nutritional deficiency
  - Gall stones
  - All of the above
8. Bariatric surgery has positive effect of which of the following condition (s)?
  - Hypertension
  - Gastro-oesophageal reflux disease
  - Sleep apnoea
  - All of the above

Next few questions are related to attitude towards bariatric surgery. You may choose to answer in a 5-point scale ranges from strongly agree to strongly disagree. (Tick inside the box as per your response).

	Strongly agree	Agree	Neutral	Disagree	Strongly disagree
I refer the patients for bariatric surgery if they fulfil the criteria					
I often suggest referral for bariatric surgery to a patient, who meets criteria for bariatric surgery, even if I see the patients due to another illness					
I feel comfortable in explaining bariatric surgery procedural options to my patients					
It is usually I who suggest bariatric surgery					
I believe that the benefits of bariatric surgery are worth the risks of the operation					
Additional continuing medical education resources in bariatric surgical care would be useful to primary care physicians in Saudi Arabia					
I feel comfortable providing care to patients who have received bariatric surgery					
I have overall positive impression of bariatric surgery as a treatment for obesity related comorbidities					

Part 3: Barriers for referring patients for bariatric surgery (Tick inside the box as per your response).

Variable	Strongly agree	Agree	Neutral	Disagree	Strongly disagree
Most of my obese patients cannot afford bariatric surgery					
Diet and exercise are effective means for sustained weight loss in morbid obese patients					
I am not comfortable managing patients after surgery					
I don’t feel confident to discuss with my patients as surgery is the treatment for morbid obese patients					
I am concerned about the risk associated with the operation (e.g. anastomosis leakage, infection, bleeding)					
I am concerned with the risk for postoperative complications					
The long-term consequences of bariatric surgery are not completely known					
The costs for bariatric surgery are a burden for society and healthcare system					

Thank you very much for completing the survey. Your participation is highly appreciated.
